# *Plasmodium falciparum* infection during dry season: IgG responses to *Anopheles gambiae* salivary gSG6-P1 peptide as sensitive biomarker for malaria risk in Northern Senegal

**DOI:** 10.1186/1475-2875-12-301

**Published:** 2013-08-30

**Authors:** André B Sagna, Lobna Gaayeb, Jean B Sarr, Simon Senghor, Anne Poinsignon, Samy Boutouaba-Combe, Anne-Marie Schacht, Emmanuel Hermann, Ngor Faye, Franck Remoue, Gilles Riveau

**Affiliations:** 1 Centre de Recherche Biomédicale Espoir Pour La Santé, 269 Route de la corniche, Sor, BP: 226, Saint-Louis, Sénégal; 2 Département de Biologie Animale, Laboratoire de parasitologie générale, Université Cheikh Anta Diop, Dakar, Sénégal; 3 CIIL, Inserm U1019, CNRS UMR 8204, Université Lille Nord de France, Institut Pasteur de Lille, 1 rue du Pr. Calmette, 59019 Lille cedex, France; 4 Institut de Recherche pour le Développement, UMR 224 MIVEGEC, 911 avenue Agropolis, BP: 64501F-34394 Montpellier, France; 5 Centre de Recherche Entomologique de Cotonou, BP: 4414, Cotonou RP 01, Bénin

**Keywords:** Dry season, *Plasmodium* infection, Anti-salivary peptide, Malaria pre-elimination, Senegal River Valley, *Anopheles* exposure

## Abstract

**Background:**

The Northern part of Senegal is characterized by a low and seasonal transmission of malaria. However, some *Plasmodium falciparum* infections and malaria clinical cases are reported during the dry season. This study aims to assess the relationship between IgG antibody (Ab) responses to gSG6-P1 mosquito salivary peptide and the prevalence of *P. falciparum* infection in children during the dry season in the Senegal River Valley. The positive association of the Ab response to gSG6-P1, as biomarker of human exposure to *Anopheles* vector bite, and *P. falciparum* infectious status (uninfected, infected-asymptomatic or infected-symptomatic) will allow considering this biomarker as a potential indicator of *P. falciparum* infection risk during the dry season.

**Methods:**

Microscopic examination of thick blood smears was performed in 371 and 310 children at the start (January) and at the end (June) of the dry season, respectively, in order to assess the prevalence of *P. falciparum* infection. Collected sera were used to evaluate IgG response to gSG6-P1 by ELISA. Association between parasitological and clinical data (infected-asymptomatic or infected-symptomatic) and the anti-gSG6-P1 IgG levels were evaluated during this period.

**Results:**

The prevalence of *P. falciparum* infection was very low to moderate according to the studied period and was higher in January (23.5%) compared to June (3.5%). Specific IgG response was also different between uninfected children and asymptomatic carriers of the parasite. Children with *P. falciparum* infection in the dry season showed higher IgG Ab levels to gSG6-P1 than uninfected children.

**Conclusions:**

The results strengthen the hypothesis that malaria transmission is maintained during the dry season in an area of low and seasonal transmission. The measurement of IgG responses to gSG6-P1 salivary peptide could be a pertinent indicator of human malaria reservoir or infection risk in this particular epidemiological context. This promising immunological marker could be useful for the evaluation of the risk of *P. falciparum* exposure observed during dry season and, by consequences, could be used for the survey of potential pre-elimination situation.

## Background

Malaria transmission in the Northern part of Senegal is low and occurs mainly between August and October due to a single rainy season from July to October [1]. This situation creates breeding sites for two members of the *Anopheles gambiae* complex (*An. gambiae s.s.* and *Anopheles arabiensis*) and for *Anopheles funestus,* which breed in fresh water and in swampy habitats with much vegetation, respectively. In this area, *Plasmodium falciparum* is virtually the only species of *Plasmodium*. The incidence of clinical cases and mortality peaks between September and November, and then rapidly declines. According to statistical data of the National Malaria Control Programme (NMCP), this region of Senegal presents the lowest national malaria attack rates, with less than five malaria declared cases per 1,000 inhabitants in 2009 [2], and could, therefore, be an interesting area of study in the scope of local malaria pre-elimination.

In several epidemiologic studies, a proportion of *P. falciparum* human carriers were found during the dry season, despite a very low level of parasite transmission [3-5]. The small numbers of clinical cases of malaria observed during the middle and at the end of the dry season are considered to be mostly due to parasitological yearly recrudescence. Some infected individuals carry gametocytes throughout the dry season, potentially inducing malaria transmission if vectors are present during this period [6]. In Northern Senegal, the construction of two dams on the Senegal River has enabled the enlargement of irrigated areas and the expansion of rice growing, providing breeding sites for mosquitoes. This situation could contribute to the maintenance of malaria transmission during the dry season [7]. The identification of *P. falciparum* carriers in human populations and the evaluation of their risks of exposure to malaria vectors are needed to carry out an effective malaria control in this particular area, considered suitable for malaria pre-elimination by the NMCP.

During its blood meal, the *Anopheles* mosquito injects saliva into the vertebrate host. The study of salivary proteins and their effects on the immune response of vertebrate host represents an innovative research tool to characterize the mechanisms of pathogens’ transmission. Therefore, the study of human-vector relationship appeared promising in the perspective of an effective control of vector-borne diseases. Numerous studies have shown that *Anopheles* salivary proteins are antigenic. In particular, it has been shown that children living in malaria-endemic areas developed IgG response to whole saliva of *An. gambiae* which was associated with the intensity of human exposure to vector bites [8]. Moreover, this specific antibody (Ab) response was predictive of malaria morbidity [8]. It was also observed that individuals with *P. falciparum* or *Plasmodium vivax* infection showed higher IgG response to *Anopheles dirus*[9] and *Anopheles darlingi* saliva [10], than uninfected individuals. In addition, one study showed that specific IgG Ab response to *An. gambiae* saliva may also be useful for assessing exposure in travellers, and thus could be a biomarker of vector exposure in individuals weakly and transiently exposed to *Anopheles* bites [11]. Altogether, the specific IgG Ab response to *Anopheles* salivary proteins represents an immunological marker of human exposure to vector bites and thus a potential indicator of the risks of *Plasmodium* transmission, as observed for various vector-borne diseases [12,13]. With the aim to develop a specific, simple and reproducible biomarker, one synthetic peptide (gSG6-P1) of the *An. gambiae* (gSG6) protein has been identified and validated as a biomarker candidate of exposure to Afro-tropical malaria vectors [14,15]. Indeed, gSG6-P1 is antigenic, highly specific to *Anopheles* and it was demonstrated that specific IgG response was especially a relevant biomarker in a context of low and seasonal exposure to malaria vectors [16-18].

This present study aimed i) to appraise the prevalence of *P. falciparum* infection during the dry season in the Senegal River valley and ii) to evaluate the association between IgG responses to gSG6-P1 salivary peptide and different clinical statuses (uninfected, asymptomatic, symptomatic) of *P. falciparum* infection in children. The final objective was to assess the potential use of the gSG6-P1 as a biomarker of *P. falciparum* infection risk during dry season.

## Methods

### Study site and population

The study was conducted in Northern Senegal, in the Senegal River Valley. This site is a typical sahelian area with less than 400 mm of rainfall per year. Malaria transmission is described as low, seasonal and occurs mainly during the rainy season between August and October with a rate of infective bites/person/night <1 [19].

A longitudinal follow-up was performed from October 2008 to January 2010 in children between one and nine years of age, in Agniam, Niandane, Pendao, Guede and Fanaye, as previously described [18,20]. In the present study, only children present at both the beginning and the end of the dry season of 2009 (January and June) were included in parasitological and immunological analyses. The characteristics of the studied population (number of children, age, and gender) are described in Table 1. Most of the study population belonged to the Fulani ethnic group.

**Table 1:** Characteristics of the studied population in the dry season, parasitological and immunological data

	**Dry season**^**a**^	**January 2009**	**June 2009**
Nb of children	681	371	310
Age, mean ± SD (years)	5.52 ± 2.5	5.27 ± 2.6	5.77 ± 2.4
Sex ratio (male/female)(%)	341/681 (50%)	188/183 (50.6%)	153/157 (49.4%)
Prevalence of *P. falciparum* infection (%)^b^	98/681 (14.4%)	87/371 (23.5%)	11/310 (3.5%)
Gametocyte carriage (%)	7/681 (1.0%)	3/371 (1.0%)	3/310 (1.0%)
% responders IgG anti-gSG6-P1^c^	102/681 (14.9%)	59/371 (15.9%)	43/310 (13.9%)

^a^Cumulative data for January and June 2009.

^b^All *P. falciparum* infection (malaria positive blood smear) with and without symptoms.

^c^Number and percentage (%) of immune responders of IgG Ab to *An. gambiae* gSG6-P1 peptide.

### Ethics statement

The present study was approved by the National Ethics Committee of the Ministry of Health of Senegal (October 2008; 0084/MSP/DS/CNRS, clinicaltrials.gov/ct2/show/NCT01545115). Oral and written informed consents were obtained from the parents or the legal guardians of the children.

### Study design

The design of the entomological, parasitological and immunological monitoring (from October 2008 to January 2010, including wet and dry seasons) of each village (Agniam, Niandane, Pendao, Guede and Fanaye) have been previously reported [18]. This present study focused on visits conducted during the dry season (January and June 2009). Exposure to *Anopheles* bites was extremely low in this area during the dry season and remained similar between villages [18]. Therefore, entomological (Human Biting Rate, HBR), immunological (percentage of IgG responders to gSG6-P1) and parasitological data (parasite prevalence and gametocyte carriage) in the present study represented the mean values of these five villages during the dry season.

### Parasitological, immunological and clinical survey

681 thick blood smears were collected during the two mentioned visits (January and June 2009) to estimate *P. falciparum* prevalence and gametocyte carriage. The slides were transferred to a nearby laboratory, stained with Giemsa and analysed under a microscope by experienced technicians. In parallel, sera collected by finger prick into BD microtainer® tubes (Franklin Lakes, New Jersey, USA) were centrifuged and 300 μL of serum was collected and stored at −20°C until use for immunological tests by ELISA. Axillary temperature of each participant was also measured.

### Study definitions

According to the *P. falciparum* malaria diagnosis and the clinical status of the disease, children were stratified in three different groups: uninfected (negative malaria blood smear), infected-asymptomatic and infected-symptomatic (positive malaria blood smear, PMBS). Children presenting a PMBS (*P. falciparum* infection) without any clinical sign, fever (axillary temperature ≥37.5°C) and/or a history of fever reported during the corresponding month, were considered infected-asymptomatic; while in the presence of fever and/or history of fever, they were classified as infected-symptomatic.

### Entomological data

Entomological data collected by human landing catches and Pyrethrum spray catches during the follow-up were previously reported and *An. gambiae s.l.* (*An. arabiensis, An. gambiae S and An. gambiae M*) have been described as the predominant species and the only vectors of *P. falciparum*[19]. Other species such as *An. funestus, Anopheles wellcomei, Anopheles pharoensis* and *Anopheles ziemanni* were also collected in low densities. At each visit, entomological sampling was performed using four adult volunteers per village (two indoors and two outdoors). Human Biting Rate (HBR) was estimated by the number of bites per person per night (BHN). It was calculated by dividing the number of *An. gambiae* caught by the total person-night for the period [19].

### Salivary peptide gSG6-P1

The gSG6-P1 peptide was designed using bioinformatics to maximize its *Anopheles* specificity and its immunogenicity, as previously described [14,15]. It was synthesized and purified (>95%) by Genepep SA (Saint Jean de Védas, France). Peptide was shipped in lyophilized form and then suspended in 0.22 μm ultra-filtered water and frozen at −20°C until use.

### Evaluation of anti-human IgG level to gSG6-P1 antigen by ELISA

ELISAs were carried out on sera to quantify IgG response to the gSG6-P1 peptide as previously described [18]. Briefly, Maxisorp plates (Nunc, Roskilde, Danemark) were coated with gSG6-P1 (20 μg/ml) in PBS (Phosphate Buffered Saline). After washing (distilled water + Tween 0.1%), each serum were incubated in duplicate at 4°C overnight at a 1/20 dilution (in PBS with 1% Tween). Mouse biotinylated Ab to human IgG (BD Pharmingen, San Diego CA, USA) was incubated at a 1/2000 dilution in PBS with 1% Tween (1 h30 at 37°C) and peroxidase-conjugated streptavidin (Amersham, les Ulis, France) was then added (1/2,000; 1 h at 37°C). Colorimetric development was carried out using ABTS (2.2′-azino-bis (3 ethylbenzthiazoline 6-sulfonic acid) diammonium; Sigma, St Louis, MO, USA) in 50 mM citrate buffer (Sigma, pH = 4, containing 0.003% H_2_O_2_) and absorbance (OD) was measured at 405 nm.

Individual results were expressed as the ΔOD value: ΔOD = ODx­ODn, where ODx represents the mean of individual optical density (OD) value in both wells with gSG6-P1 antigen and ODn the individual OD value for each serum without gSG6-P1 antigen. Specific anti-gSG6-P1 IgG response were also assayed in non-*Anopheles* exposed individuals (n = 12 – neg; North of France) in order to quantify the non-specific background Ab level and to calculate the specific immune response threshold: TR = mean (∆ODneg) + 3SD = 0.180. An exposed individual was then classified as an immune responder (IR) if its ΔOD > 0.180.

### Statistical analysis

Data were analysed with Graph Pad Prism® (Graph Pad Software, San Diego, USA). Chi^2^ test was used to compare *P. falciparum* prevalence between January and June, and also between IgG immune responders to gSG6-P1 and non-responders. Mann–Whitney U test was used for the comparison of IgG levels between uninfected and infected-asymptomatic or infected-symptomatic children. The same test was used to compare differences between uninfected and infected children. Differences of IgG response levels between more than three groups were calculated using the non parametric Kruskal-Wallis test. The association between *P. falciparum* infection and immune responders to gSG6-P1 peptide was calculated using the relative risk (RR), and 95% confidence intervals (CI) were calculated by the Miettinen method. All differences were considered significant at p < 0.05.

## Results

### Malaria infection during the dry season

As indicated in Table 1, 681 children have been included during the dry season (371 in January 2009 and 310 in June 2009) among them 304 were present at both visits. Mean age and sex ratio were similar at both visits. Microscopic examination of thick blood smears revealed some *P. falciparum* infection in children during dry season. The prevalence of *P. falciparum* infection was higher in January 2009 (23.5%) than in June 2009 (3.5%) (p < 0.001, Chi^2^ Test). No difference in *P. falciparum* infection was observed between children under 5 years of age and the older ones (Additional file 1). Only nine clinical cases of malaria were recorded during both visits, six in January 2009 (1.6%) and three in June 2009 (1%). Gametocyte carriage during this period remained very low (1%). HBR was also very low and was estimated at 0.3 during the cold dry season (January 2009) and 1.52 during the hot dry season (June 2009) [18]. This low exposure to *Anopheles* bites was confirmed by immunological data that showed 14.9% of IgG responders to gSG6-P1 (Table 1). In addition, no infected *Anopheles* were collected during this dry season in the area (Entomological Inoculation Rate, EIR = 0) [19].

### IgG response levels to gSG6-P1 according to malaria infection

**Figure 1:**
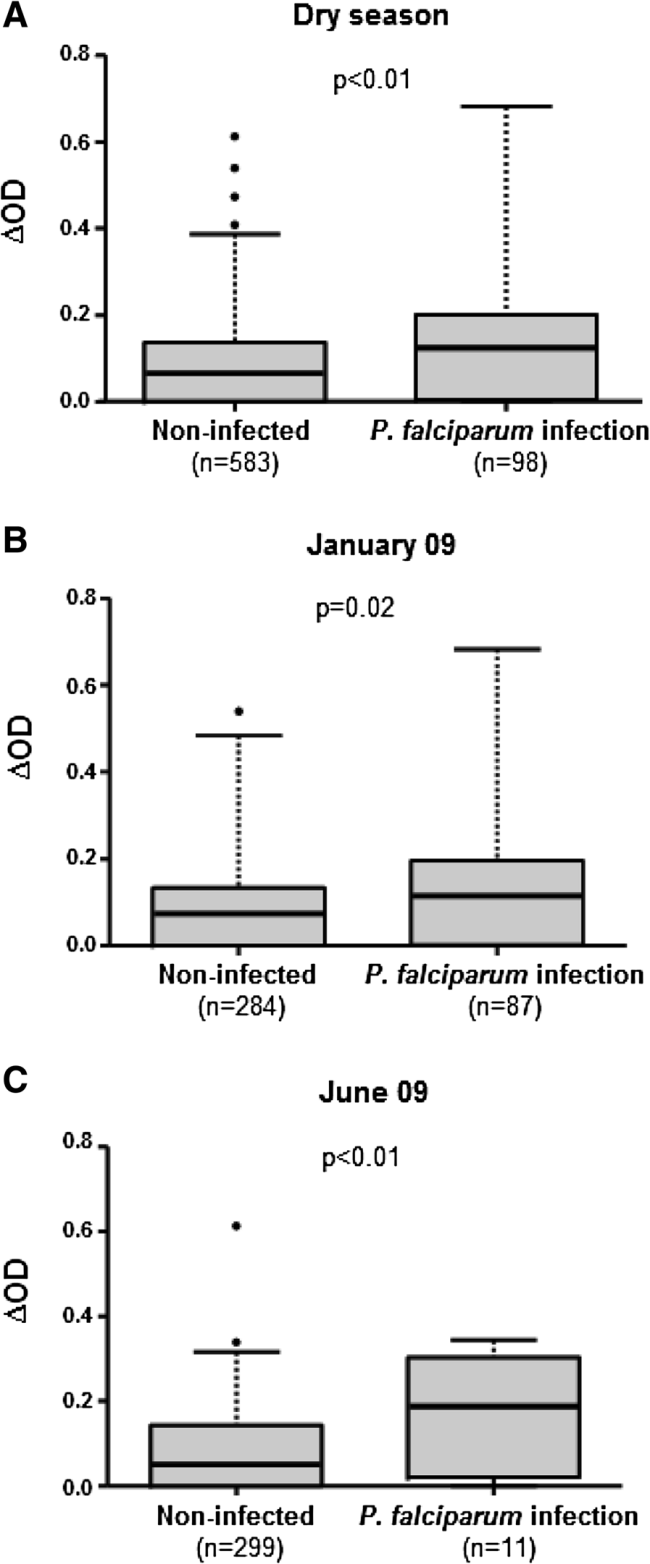
**IgG response levels to gSG6-P1 peptide from non-infected and infected children.** Box plots indicate IgG response (∆OD) values to gSG6-P1 peptide from uninfected individuals and children with *P. falciparum* infection during the whole dry season (cumulative data) **(A)**, in January 09 **(B)** and in June 09 **(C)**. Boxes display the median ∆OD value, 25^th^ and 75^th^ percentiles. The whiskers show the 5^th^/95^th^ percentiles and the dots indicate the outliers. The P value was determined according to the Mann–Whitney U test.

First, differences of specific IgG response levels to gSG6-P1 salivary peptide were compared between individuals with *P. falciparum* infection (children who presented a PMBS, infected-symptomatic or infected-asymptomatic) and uninfected. Comparison showed that the median of specific IgG Ab level was significantly higher in *P. falciparum*-infected children compared to uninfected ones during the dry season (p < 0.01, Figure 1A, Mann–Whitney U Test). A similar pattern of specific IgG level according to malaria infection was observed in January (p = 0.02, Figure 1B) and in June (p < 0.01, Figure 1C).

### IgG response levels to gSG6-P1 according to malaria status

**Figure 2:**
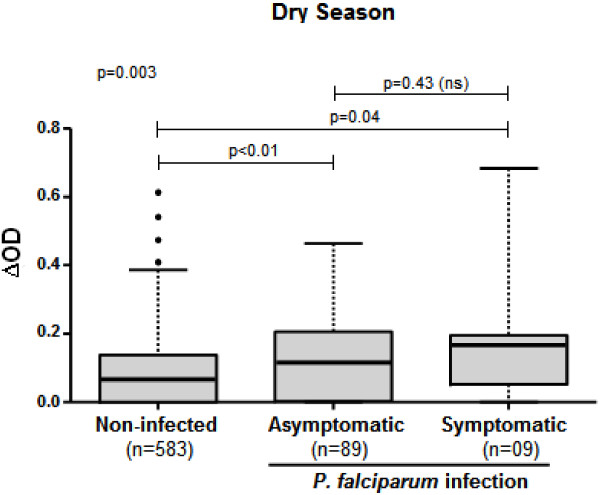
**IgG response levels to gSG6-P1 peptide according to malaria status.** Box plots show anti-gSG6-P1 IgG level (∆OD) between uninfected, infected-asymptomatic and infected-symptomatic individuals (cumulative data from January and June 2009). Boxes display the median ∆OD value, 25^th^ and 75^th^ percentiles. The whiskers show the 5^th^/95^th^ percentiles and the dots indicate the outliers. Differences between two or three groups were tested using Mann Whitney test and Kruskal Wallis test, respectively.

The specific IgG Ab response to gSG6-P1 was then analysed according to the three different statuses of *P. falciparum* infection (uninfected, infected-asymptomatic and infected-symptomatic) during the dry season (Figure 2; cumulative data for January and June 2009). The anti-gSG6-P1 IgG levels were significantly different according to the status of *P. falciparum* infection (p = 0.003, Kruskal-Wallis Test). Indeed, specific IgG responses were significantly higher in both infected-asymptomatic and infected-symptomatic children compared to uninfected ones (p < 0.01 and p = 0.04, Mann–Whitney U test, respectively). But, no statistical difference was observed in IgG Ab responses between infected-asymptomatic and infected-symptomatic children, (p = 0.43, Mann–Whitney U test). Similar results were obtained when data were analysed separately in January and in June 2009 (Additional file 2). IgG response levels remained higher in infected-asymptomatic children compared to uninfected ones (p < 0.05). Nevertheless, there was no difference either between uninfected and infected-symptomatic or between infected-asymptomatic and infected-symptomatic.

### IgG response levels to gSG6-P1 and *P. falciparum* infection risk

**Figure 3:**
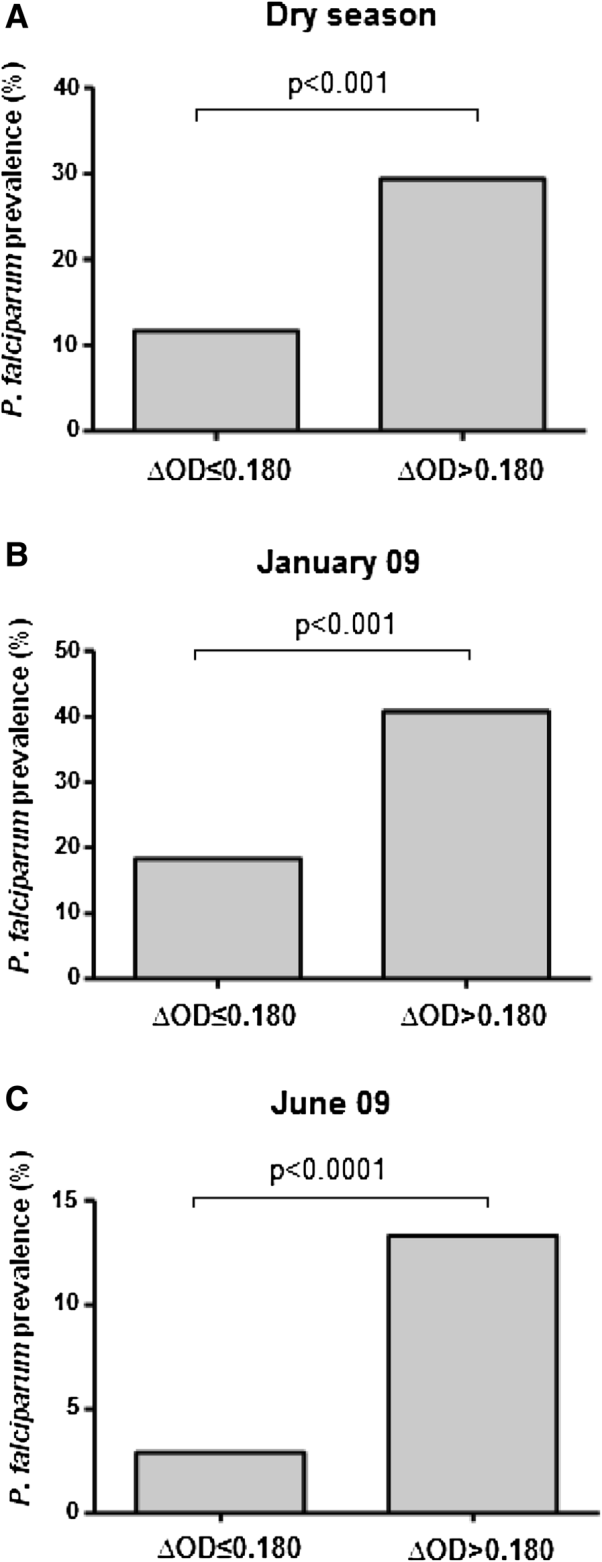
***Plasmodium falciparum*prevalence between IgG responders and non-responders to gSG6-P1 peptide.** Data present the rate of *P. falciparum* infection according to the two defined immune responder groups: non-responders to gSG6-P1 (∆OD ≤ 0.180) and responders to gSG6-P1 (∆OD > 0.180) during the whole dry season (cumulative data) **(A)**, in January 09 **(B)** and in June 09 **(C)**. Statistical significant difference between these two groups is indicated using a Chi^2^ test.

To examine if the evaluation of specific IgG Ab levels to gSG6-P1 could be a suitable method to estimate the probability of *P. falciparum* infection during the dry season, i.e. under conditions of low transmission, prevalence of *P. falciparum* infection was compared between immune IgG responders to gSG6-P1 (∆OD > 0.180) and non-immune responders (∆OD ≤ 0.180) (Figure 3). Positive and significant association between malaria infection prevalence and immune responder to gSG6-P1 was observed. Indeed, the risk of *P. falciparum* infection was 2–7 times higher in immune responders than in non-responders during the dry season (Figure 3A; relative risk [RR] = 2.504, 95% CI: 1.70-3.67, p < 0.001, Chi^2^ test), in January (Figure 3B; RR = 2.014, 95% CI: 1.34-3.00, p < 0.001) and in June (Figure 3C; RR = 7.451, 95% CI: 3.08-17.97, p < 0.0001). Taken together, these data suggest that positive IgG response to gSG6-P1 could be a pertinent and reliable indicator for suspected *P. falciparum* infection in individuals living in a low transmission context.

## Discussion

In the first part of the study, *P. falciparum* infection in the human population during the dry season was investigated in the Senegal River Valley reported as a low malaria transmission setting [19]. *P. falciparum* infection was observed during the dry season and was higher in January (23.5%) than in June (3.5%). This finding confirmed previous studies in a neighbouring area [21,22] in which a moderate *P. falciparum* infection rate decreases throughout the dry season (December to June) [18].

The paradox observed in this area, between low or no transmission (EIR = 0) and high or moderate malaria parasite prevalence, has previously been described in a neighbouring area [21] and elsewhere in Africa [23-25]. Such an observation raises again the question about the relationships between the EIR indicator and its pertinence, and the prevalence of malaria infection. The absence of infection in *Anopheles* could be explained by a lack of sensitivity of entomological sampling methods used, in a special context where a very small number of *An. gambiae s.l.* is collected. Indeed, *An. gambiae s.s and An. arabiensis* were already proven to be highly anthropophilic and predominant malaria vectors in the Senegal River Valley [19]. *Anopheles* infection rate of 1.37% was observed one year later during the dry season [19]. This suggests that malaria transmission could occur during the dry season between November and June corresponding to off-season agriculture. However, its intensity could differ from one year to another [1]. Thus, irrigated fields’ crops, annual flood of the river, temporary ponds or ditches could provide breeding sites for remaining *Anopheles*[19,26,27], and thereby contribute to malaria transmission during the dry season. However, it should be noticed that very few clinical cases of malaria were recorded in this area during the dry season. Indeed, the Senegalese NMCP reported in 2009 less than five malaria cases per 1,000 inhabitants of the River Valley [2]. In our study, most of infected children were asymptomatic carriers of the parasite and the identification of such individuals could be very relevant for a better malaria control, especially during the dry season and in areas of malaria pre-elimination.

In this regard, the present study shows that IgG response to gSG6-P1 was higher in infected-asymptomatic children than in uninfected ones. Statistical significance between infected-asymptomatic and uninfected children was stronger (p < 0.01) when data were cumulated (January and June 2009); probably due to the huge amount of overlap between groups during these two visits. However, IgG response to gSG6-P1 peptide remained significantly higher in infected-asymptomatic children than in uninfected ones when data were analysed separately in January (p = 0.04) and in June (p = 0.02). So, IgG response to *An. gambiae* gSG6-P1 salivary peptide could be a pertinent biomarker of *P. falciparum* asymptomatic infection. Such a distinction of asymptomatic *P. falciparum* infected individuals compared to uninfected children appears considerable for malaria control in low endemicity areas, and especially during the dry season. Indeed, most of infected children are asymptomatic carriers of the parasite and long-term asymptomatic carriage of *P. falciparum* throughout the dry season is commonly observed [3,4,28]. Infected-asymptomatic individuals could serve as a crucial reservoir for the parasite and could transmit *P. falciparum* to uninfected *Anopheles* vectors which remain after the rainy season, contributing to the maintenance of malaria transmission in the dry season and its re-starting after the first rains [5,29]. Therefore, monitoring the IgG response to gSG6-P1 salivary peptide as a biomarker of *P. falciparum* asymptomatic infection could be used as a valuable tool in a malaria pre-elimination context.

In addition, the present study showed that high IgG levels to gSG6-P1 could be predictive indicators of *P. falciparum* infection. This result is in accordance with previous results in Senegal showing that children with subsequently clinical *P. falciparum* malaria presented higher anti-saliva IgG response than did non-infected children [8]. In addition, Andrade *et al.*[10] have showed that higher anti-*An. darlingi* saliva Ab response could indicate human exposure to *P. vivax* and biomarker of *P. vivax* in Brazil. Recently, a study in Burkina-Faso showed a positive correlation between malaria incidence and IgG response against the recombinant protein of *An. gambiae* (gSG6) [30]. In Kenya, it has also been indicated that parasite prevalence was associated with the increase of anti-gSG6-P1 IgG prevalence [31]. Altogether, these results suggest that anti-saliva (or protein/peptide) Ab response, previously demonstrated to be biomarker of exposure to *Anopheles* bites in numerous contexts, could also be a useful indicator of malaria infection and/or morbidity risks in this particular context. It is quite conceivable that salivary peptides could complete in the near future, routine thick blood smears performed in the framework of active case detection of malaria infection or in the evaluation of vector control strategies. Indeed, it was shown that it was possible to assess by ELISA, IgG response to gSG6-P1 on eluates of blood drops collected on blotting paper [16,32,33]. This sampling technique could be performed concomitantly to thick blood smears and would allow making several molecular analyses (DNA sequencing of organisms, modern diagnostic techniques based on biomarkers detection.) on the same sample. This method is therefore suitable for multidisciplinary large-scale studies [34]. Furthermore, it is also conceivable to develop such immunological biomarker by the use of a simple self-test strip, like rapid diagnostic test (RDT). This would rapidly identify people at risk or even asymptomatic malaria infected individuals and begin early treatment. Nevertheless, the use of biomarkers of human exposure to *Anopheles* bites as an immunological indicator of disease risk remains to be largely studied. For example, there is a lack of a conventional threshold of immune response to salivary proteins/peptide at which an individual is at risk of malaria infection. The intensity of exposure or transmission varies according to the area and season. Despite these limitations, data of the current study indicate the potentiality to use biomarkers of human exposure to vectors as a reliable and useful tool to detect malaria human reservoirs and help predicting malaria risk, and this in a context of very low exposure to vectors, such as malaria pre-elimination settings. This point appeared to be clearly relevant in regard to the observed results in June (end of dry season with very low exposure to *Anopheles* bites) where the risk of *P. falciparum* infection was seven times higher in immune responders than in non-responders.

A limitation of this study could be linked to an under-evaluation of *P. falciparum* infection prevalence during the dry season, since it was only determined by microscopy examination. However, this gold standard technique for malaria screening has limited sensitivity for detection of very low parasite densities. Indeed, it has been shown that, in areas with low or very low intensity of malaria transmission, sub-microscopic infections were commonly seen [35,36]. It would be important to improve this detection using more sensitive methods such as gene amplification by Polymerase Chain Reaction (PCR) [37-39]. Nevertheless, the presence of *P. falciparum* infection has been revealed with microscopic detection, suggesting that malaria transmission occurs during the dry season in this area.

## Conclusions

During the low-transmission season (dry season), *P. falciparum* infections were observed in the Northern Senegal River Valley. Parasitological data have been correlated with anti-gSG6-P1 IgG responses, indicating that this *Anopheles* salivary-antigen specific response could be a pertinent epidemiological biomarker of *P. falciparum* exposure during the dry season. Furthermore, the analysis revealed that high levels of gSG6-P1 specific IgG response could be used as an indicator of infection risk and even to discriminate uninfected and infected-asymptomatic carriers of the parasite during the dry season. This biomarker could be very useful for national malaria control programs to identify asymptomatic carriers of *P. falciparum* in low and seasonal transmission areas and, by consequences, in a context of malaria pre-elimination.

## Competing interests

The authors declare that they have no competing interests.

## Authors’ contributions

ABS participated in field surveys, carried out the immunological assessments, analysed data and drafted the manuscript. JBS, LG, SS, MP, AMS, EH, NF participated in study coordination, field surveys, data collection and microscopic examination. SBC participated to data analysis. GR and FR participated in study design, preparation and writing of the manuscript. All authors contribute to revision the manuscript and read and approved the final version.

## Supplementary Material

Additional file 1 **Prevalence of*P. falciparum* infection during the dry season, stratified by age group.** The table summarizes *P. falciparum* infection between children under 5 years of age and the older ones during the dry season. Click here for file

Additional file 2 **IgG response levels to gSG6-P1 peptide according to malaria status.** Box plots show gSG6-P1 specific IgG response levels (∆OD) according to three *P. falciparum* infection statuses. Boxes display the median ∆OD value, 25^th^ and 75^th^ percentiles. The whiskers show the 5^th^/95^th^ percentiles and the dots indicate the outliers. Differences between two or three groups were tested using Mann Whitney test and Kruskal Wallis test, respectively. Click here for file
