# Supplementary material for: Plasmodium falciparum infection during dry season: IgG responses to Anopheles gambiae salivary gSG6-P1 peptide as sensitive biomarker for malaria risk in Northern Senegal
Source: Malar J. 2013 Aug 30;12:301. doi: 10.1186/1475-2875-12-301 (PMC3766161; doi:10.1186/1475-2875-12-301)
Supplement: Additional file 1 — Prevalence ofP. falciparum infection during the dry season, stratified by age group. The table summarizes P. falciparum infection between children under 5 years of age and the older ones during the dry season. [file 1475-2875-12-301-S1.doc]

**Additional file 1 Prevalence of *P. falciparum* infection during the dry season, stratified by age group**

|  | **Dry season^a^** | **January 2009** | **June 2009** |
| --- | --- | --- | --- |
| No. of children | 681 | 371 | 310 |
| No of positive (%)^b^ | 98 (14.4) | 87 (23.5) | 11 (3.5) |
| No of positive children by age group (%) |  |  |  |
| [1-5[ | 48/275 (17.5) | 43/159 (27.0) | 5/116 (4.3) |
| [5-9] | 50/406 (12.3) | 44/212 (20.7) | 6/194 (3.1) |

^a^Cumulative data for January and June 2009

^b^All *P. falciparum* infection (malaria positive blood smear) with and without symptoms
