# Supplementary figures and images for: Plasmodium falciparum infection during dry season: IgG responses to Anopheles gambiae salivary gSG6-P1 peptide as sensitive biomarker for malaria risk in Northern Senegal
Source: Malar J. 2013 Aug 30;12:301. doi: 10.1186/1475-2875-12-301 (PMC3766161; doi:10.1186/1475-2875-12-301)

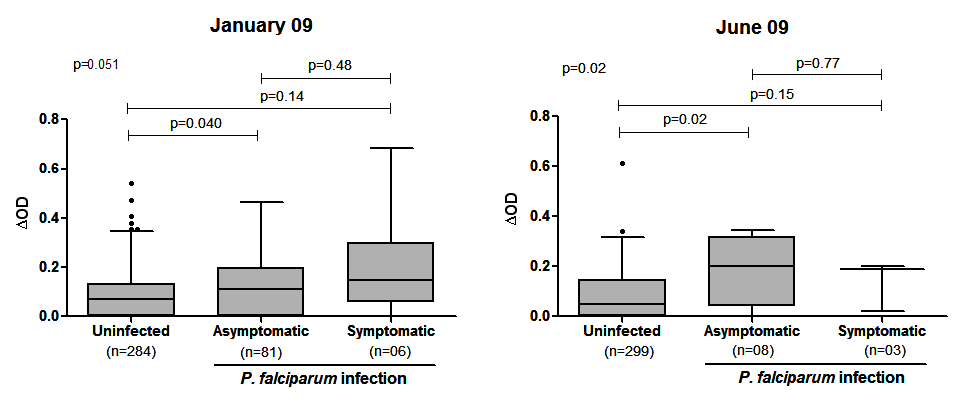

Supplement: Additional file 2 — IgG response levels to gSG6-P1 peptide according to malaria status. Box plots show gSG6-P1 specific IgG response levels (∆OD) according to three P. falciparum infection statuses. Boxes display the median ∆OD value, 25th and 75th percentiles. The whiskers show the 5th/95th percentiles and the dots indicate the outliers. Differences between two or three groups were tested using Mann Whitney test and Kruskal Wallis test, respectively. [file 1475-2875-12-301-S2.tiff]
